# Supplementary material for: Effects of an oral exercise intervention on pre-frailty or frailty in older people: a randomized clinical trial
Source: Commun Med (Lond). 2026 Jan 9;6:96. doi: 10.1038/s43856-025-01361-0 (PMC12886859; doi:10.1038/s43856-025-01361-0)
Supplement: Supplementary file 2 — Supplementary Information [file 43856_2025_1361_MOESM2_ESM.pdf]

Supplementary Table 1. Participants' characteristics (intention-to-treat analysis, Complete Version).

Table caption: This table summarizes baseline demographic, clinical, physical, oral function, and nutritional characteristics of participants in the four oral exercise frequency groups under the intention-to-treat analysis. Continuous variables are presented as mean ± standard deviation, and categorical variables as number (percentage). p-values are based on comparisons among groups. Bold variables indicate the main outcome measures: Number of frailty criteria, SMI, Number of present teeth, Number of functional teeth, Number of oral hypofunction criteria, MNA, Social frailty score, and GDS15.

Variables	Total		3 times/day & everyday (n=14)		3 times/day & 3 days/week (n=15)		once/day & everyday (n=14)		once/day & 3 days/week (n=15)		p value ‡
		95% CI		95% CI		95% CI		95% CI		95% CI	
age (y)	79.71±6.39*	(78.026, 81.388)	76.86±4.75	(74.113, 79.602)	78.87±5.00	(76.099, 81.635)	81.64±6.05	(78.152, 85.134)	81.40±8.42	(76.739, 86.061)	0.147
gender (Male)	12 (20.69)†	(11.611, 32.146)	3 (21.43)	(4.911, 70.788)	4 (26.67)	(7.805, 55.127)	2 (14.28)	(1.772, 42.843)	3 (20.00)	(4.303, 48.099)	0.877
**Number of frailty criteria**	2.17±0.99	(1.911, 2.434)	2.07±1.00	(1.496, 2.647)	2.13±0.83	(1.672, 2.595)	2.36±1.28	(1.62, 3.095)	2.13±0.92	(1.626, 2.64)	0.949
Grip strength (kg)	18.73±6.39	(17.047, 20.405)	19.12±4.10	(16.756, 21.486)	20.82±7.46	(16.69, 24.95)	15.79±7.23	(11.611, 19.96)	19.01±5.72	(15.841, 22.172)	0.207
Walking speed (m/s)	1.04±0.27	(0.965, 1.107)	1.03±0.25	(0.883, 1.168)	1.10±0.25	(0.956, 1.235)	1.04±0.32	(0.856, 1.227)	0.98±0.27	(0.83, 1.134)	0.659
**SMI (kg/m^2)**	6.13±0.81	(5.908, 6.351)	5.99±0.60	(5.63, 6.359)	6.34±0.80	(5.879, 6.799)	6.05±0.94	(5.477, 6.619)	6.12±0.91	(5.597, 6.645)	0.658
BMI (kg/m^2)	22.24±4.13	(21.113, 23.368)	21.28±5.13	(18.317, 24.236)	23.44±3.01	(21.777, 25.112)	21.42±3.22	(19.558, 23.275)	23.31±4.56	(20.786, 25.837)	0.191
Skeletal muscle mass	33.45±6.59	(31.655, 35.251)	32.64±4.94	(29.781, 35.491)	34.81±6.73	(31.087, 38.539)	32.73±7.18	(28.394, 37.068)	33.81±7.09	(29.886, 37.741)	0.750
Appendicular skeletal muscle mass	14.44±3.17	(13.571, 15.299)	14.09±2.48	(12.592, 15.593)	15.19±3.28	(13.291, 17.08)	14.21±3.89	(11.859, 16.564)	14.21±3.10	(12.422, 16)	0.757
Number of present teeth (n)	19.88±7.47	(17.916, 21.843)	20.50±7.69	(16.058, 24.942)	19.8±7.70	(15.535, 24.065)	19.79±6.23	(16.189, 23.382)	19.47±8.72	(14.635, 24.298)	0.955
Number of functional teeth (n)	26.52±3.24	(25.665, 27.369)	25.93±2.23	(24.638, 27.219)	27.07±2.15	(25.874, 28.259)	25.36±5.27	(22.314, 28.401)	27.60±2.10	(26.438, 28.762)	0.307
**Number of Oral hypofunction criteria**	3.50±1.41	(3.131, 3.869)	3.00±1.11	(2.359, 3.641)	3.40±1.40	(2.622, 4.178)	4.14±1.83	(3.084, 5.202)	3.47±1.06	(2.88, 4.054)	0.200
Bacterial count on the dorsal surface of the tongue (10^6)(cfu/ml)	6.11±6.52	(4.396, 7.824)	5.86±5.65	(2.593, 9.12)	5.81±8.06	(1.348, 10.276)	6.57±7.14	(2.449, 10.694)	6.21±5.54	(3.146, 9.279)	0.757
Oral moisture status	25.73±3.55	(24794, 26.659)	25.93±2.95	(24.227, 27.63)	25.06±5.45	(22.044, 28.076)	25.77±2.62	(24.259, 27.283)	26.16±2.52	(24.766, 27.561)	0.954
Maximum occlusal force (N)	468.49±319.14	(384.576, 552.401)	487.25±345.84	(287.566, 686.934)	437.76±282.77	(281.173, 594.354)	394.88±309.85	(215.979, 573.785)	550.4±347.16	(358.151, 742.656)	0.519
Tongue and lip motor function											
/pa/ sound (times/s)	5.71±0.96	(5.455, 5.962)	5.58±1.16	(4.907, 6.25)	6.01±0.79	(5.576, 6.45)	5.47±1.08	(4.85, 6.093)	5.75±0.81	(5.296, 6.197)	0.478
/ta/ sound (times/s)	5.61±0.98	(5.354, 5.870)	5.51±1.13	(4.86, 6.168)	5.91±0.65	(5.549, 6.265)	5.40±1.13	(4.746, 6.054)	5.61±0.99	(5.059, 6.154)	0.779
/ka/ sound (times/s)	5.24±0.96	(4.992, 5.496)	5.35±1.04	(4.751, 5.949)	5.44±0.68	(5.062, 5.811)	5.03±1.02	(4.442, 5.615)	5.15±1.11	(4.541, 5.765)	0.736
Maximum tongue pressure (kPa)	22.44±9.00	(20.074, 24.809)	25.34±7.5	(21.006, 29.666)	24.8±10.32	(19.086, 30.514)	17.96±7.25	(13.776, 22.153)	21.56±9.34	(16.388, 26.732)	0.095
Masticatory function (mg/dL)	211.58±85.28	(189.159, 234.005)	209.39±95.28	(154.382, 264.404)	227.3±106.98	(168.054, 286.546)	185.09±69.31	(145.073, 225.106)	222.63±64.58	(186.868, 258.398)	0.554
EAT10	4.07±5.67	(2.567, 5.574)	2.36±3.95	(0.074, 4.64)	4.67±7.35	(0.599, 8.734)	6.00±6.46	(2.272, 9.728)	3.21±3.81	(1.017, 5.412)	0.521
RSST (times)	3.09±1.84	(2.603, 3.569)	3.57±2.38	(2.199, 4.944)	2.80±1.82	(1.792, 3.808)	3.14±1.29	(2.397, 3.889)	2.87±1.81	(1.866, 3.868)	0.618
**MNA**	24.11±3.89	(23.088, 25.136)	23.86±3.24	(21.988, 25.726)	24.83±3.96	(22.639, 27.028)	23.68±4.90	(20.851, 26.506)	24.03±3.63	(22.022, 26.045)	0.755
**Social frailty score**	1.98±1.22	(1.662, 2.304)	1.50±0.65	(1.124, 1.876)	1.60±1.12	(0.979, 2.221)	2.29±1.27	(1.554, 3.017)	2.53±1.46	(1.726, 3.34)	0.142
**GDS15**	4.28±3.52	(3.351, 5.201)	3.00±2.32	(1.66, 4.34)	3.80±2.93	(2.176, 5.424)	4.36±3.59	(2.286, 6.428)	5.87±4.50	(3.374, 8.36)	0.322
Stroke	1 (1.72)	(0.041, 9.178)	0 (0.00)	(0.000, 19.513)	0 (0.00)	(0.000, 19.513)	0 (0.00)	(0.000, 19.513)	1 (6.67)	(0.168, 31.945)	0.405
Heart disease	5 (8.62)	(2.817, 19.261)	1 (7.14)	(0.181, 33.667)	2 (13.33)	(1.667, 38.570)	1 (7.14)	(0.181, 33.667)	1 (6.67)	(0.168, 31.945)	0.903
Respiratory disease	5 (8.62)	(2.817, 19.261)	3 (21.43)	(4.879, 50.721)	1 (6.67)	(0.168, 31.945)	1 (7.14)	(0.181, 33.667)	0 (0.00)	(0.000, 20.581)	0.218
Hypertension	25 (43.10)	(30.740, 56.329)	5 (35.71)	(12.939, 64.900)	5 (33.33)	(11.808, 61.615)	7 (50.00)	(23.025, 76.975)	8 (53.33)	(26.595, 78.744)	0.613
Diabetes mellitus	7 (12.07)	(5.002, 23.292)	5 (35.71)	(12.939, 64.900)	0 (0.00)	(0.000, 19.513)	0 (0.00)	(0.000, 19.513)	2 (13.33)	(1.667, 38.570)	0.010
Kidney disease	2 (3.45)	(0.419, 11.982)	2 (14.29)	(1.772, 42.843)	0 (0.00)	(0.000, 19.513)	0 (0.00)	(0.000, 19.513)	0 (0.00)	(0.000, 20.581)	0.089
Knee osteoarthritis	4 (6.90)	(1.885, 17.059)	1 (7.14)	(0.181, 33.667)	1 (6.67)	(0.168, 31.945)	1 (7.14)	(0.181, 33.667)	1 (6.67)	(0.168, 31.945)	1.000
Osteoporosis	10 (17.24)	(8.602, 29.733)	4 (28.57)	(8.428, 58.074)	1 (6.67)	(0.168, 31.945)	3 (21.43)	(4.879, 50.721)	2 (13.33)	(1.667, 38.570)	0.429
Rheumatoid arthritis	4 (6.90)	(1.885, 17.059)	1 (7.14)	(0.181, 33.667)	0 (0.00)	(0.000, 19.513)	2 (14.28)	(1.772, 42.843)	1 (6.67)	(0.168, 31.945)	0.512
Parkinson's disease	2 (3.45)	(0.419, 11.982)	0 (0.00)	(0.000, 19.513)	0 (0.00)	(0.000, 19.513)	1 (7.14)	(0.181, 33.667)	1 (6.67)	(0.168, 31.945)	0.557
Anemia	4 (6.90)	(1.885, 17.059)	1 (7.14)	(0.181, 33.667)	2 (13.33)	(1.667, 38.570)	0 (0.00)	(0.000, 19.513)	1 (6.67)	(0.168, 31.945)	0.571
Hearing dysfunction	1 (1.72)	(0.041, 9.178)	0 (0.00)	(0.000, 19.513)	1 (6.67)	(0.168, 31.945)	0 (0.00)	(0.000, 19.513)	0 (0.00)	(0.000, 20.581)	0.405

*: average±standard deviation, †: n (%), ‡: p-values for group comparisons using Kruskal–Wallis test for continuous variables and chi-square test for categorical variables

CI: Confidence Interval, BMI: Body Mass Index, SMI: Skeletal Mass Index, EAT10: The 10-item Eating Assessment Tool, RSST: Repetitive Salive Swallowing Test, MNA: Mini Nutritional Assessment, GDS: Geriatric depression scale

Supplementary Table 2. The baseline and follow-up outcomes and minimum clinically important difference in the each groups (intention-to-treat analysis, Complete Version).

Table caption: This table presents baseline and follow-up values for frailty criteria, physical performance, sarcopenia-related measures, oral function, and nutritional status in four oral exercise frequency groups under the intention-to-treat analysis. It also includes p-values, mean differences with 95% confidence intervals, and minimum clinically important differences (MCID) for each outcome. Bold variables indicate the main outcome measures: Number of frailty criteria, SMI, Number of present teeth, Number of functional teeth, Number of oral hypofunction criteria, MNA, Social frailty score, and GDS15.

	3 times/day & everyday (n=14)					3 times/day & 3 days/week (n=15)					once/day & everyday (n=14)					once/day & 3 days/week (n=15)					p-value[†]
Variables	baseline	Follow-Up	p-value[*]	Mean difference (CI)	MCID	baseline	Follow-Up	p-value	Mean difference (CI)	MCID	baseline	Follow-Up	p-value	Mean difference (CI)	MCID	baseline	Follow-Up	p-value	Mean difference (CI)	MCID	
**Number of frailty criteria**	2.07±1.00[‡]	1.71±0.91	0.096	-0.357 (-0.787, 0.073)[§]	0.37	2.13±0.83	1.53±0.99	0.070	-0.600 (-1.255, 0.055)	0.59	2.36±1.28	1.79±1.25	0.155	-0.571 (-1.379, 0.236)	0.70	2.13±0.92	1.53±1.25	0.013	-0.600 (-1.008, -0.192)	0.37	0.949
Grip strength (kg)	19.12±4.10	19.41±3.24	0.345	0.293 (-2.203, 2.788)[‖]	1.21	20.82±7.46	21.09±7.66	0.755	0.273 (-1.949, 2.496)	1.14	15.79±7.23	16.35±7.68	0.414	0.564 (-1.543, 2.672)	1.02	19.01±5.72	18.55±5.04	0.211	-0.453 (-2.198, 1.292)	0.89	0.207
Walking speed (m/s)	1.03±0.25	1.03±0.35	0.917	0.006 (-0.166, 0.179)	0.08	1.1±0.25	1.15±0.47	0.460	0.051 (-0.249, 0.35)	0.15	1.04±0.32	1.02±0.27	0.799	-0.017 (-0.170, 0.135)	0.07	0.98±0.27	1.02±0.38	0.280	0.035 (-0.292, 0.362)	0.17	0.659
**SMI (kg/m^2)**	5.99±0.60	5.98±0.47	0.308	-0.017 (-0.378, 0.345)	0.17	6.34±0.8	6.42±0.72	0.152	0.083 (-0.140, 0.306)	0.11	6.05±0.94	6.02±0.94	0.508	-0.027 (-0.157, 0.102)	0.06	6.12±0.91	6.2±0.75	0.730	0.021 (-0.458, 0.499)	0.23	0.658
BMI (kg/m^2)	21.28±5.13	21.07±5.09	0.055	-0.202 (-0.848, 0.444)	0.31	23.44±3.01	23.49±2.99	0.691	0.042 (-0.686, 0.769)	0.37	21.42±3.22	21.19±3.04	0.047	-0.231 (-0.610, 0.148)	0.18	23.31±4.56	23.23±4.44	0.650	-0.081 (-0.614, 0.452)	0.27	0.191
Skeletal muscle mass	32.64±4.94	32.68±4.65	0.824	0.043 (-1.074, 1.159)	0.54	34.81±6.73	34.83±6.1	0.382	0.020 (-1.382, 1.422)	0.72	32.73±7.18	32.64±7.17	0.646	-0.092 (-0.656, 0.472)	0.26	33.81±7.09	33.8±6.51	0.842	-0.013 (-1.423, 1.397)	0.72	0.750
Appendicular skeletal muscle mass	14.09±2.48	14.04±2.13	0.455	-0.050 (-0.861, 0.761)	0.37	15.19±3.28	15.37±3.1	0.124	0.182 (-0.475, 0.840)	0.32	14.21±3.89	14.16±3.89	0.678	-0.050 (-0.417, 0.317)	0.17	14.21±3.1	14.31±2.58	0.730	-0.100 (-1.382, 1.182)	0.62	0.757
**Number of present teeth (n)**	20.5±7.69	20.43±7.76	0.317	-0.071 (-0.347, 0.204)	0.13	19.8±7.7	19.8±7.7	1.000	0.000 (0.000, 0.000)	0.00	19.79±6.23	19.79±6.23	1.000	0.000 (0.000, 0.000)	0.00	19.47±8.72	19.47±8.72	1.000	0.000 (0.000, 0.000)	0.00	0.955
**Number of functional teeth (n)**	25.93±2.23	25.86±2.18	0.317	-0.071 (-0.347, 0.204)	0.13	27.07±2.15	27.07±2.15	1.000	0.000 (0.000, 0.000)	0.00	25.36±5.27	25.36±5.27	1.000	0.000 (0.000, 0.000)	0.00	27.6±2.1	27.6±2.1	1.000	0.000 (0.000, 0.000)	0.00	0.307
**Number of Oral hypofunction criteria**	3±1.11	2.5±1.16	0.070	-0.500 (-1.468, 0.468)	0.47	3.4±1.4	3.07±1.49	0.374	-0.333 (-1.596, 0.929)	0.65	4.14±1.83	3.5±1.87	0.084	-0.643 (-1.958, 0.672)	0.64	3.47±1.06	2.53±1.13	0.003	-0.933 (-1.873, 0.007)	0.48	0.200
Bacterial count on the dorsal surface of the tongue (10^6)(cfu/ml)	5.86±5.65	5.72±4.35	0.969	-0.139 (-4.07, 3.79)	1.91	5.81±8.06	6.49±7.97	0.600	0.679 (-6.524, 7.882)	3.68	6.57±7.14	9.44±8.39	0.093	2.865 (-5.292, 11.022)	3.96	6.21±5.54	11.63±16.35	0.307	5.417 (-9.235, 20.068)	7.49	0.757
Oral moisture status	25.93±2.95	29.07±1.01	0.005	3.139 (0.257, 6.022)	1.40	25.06±5.45	27.97±2.15	0.088	2.910 (-2.859, 8.679)	2.95	25.77±2.62	27.69±2.68	0.047	1.921 (-1.311, 5.153)	1.57	26.16±2.52	29.19±2.01	0.003	3.030 (0.646, 5.414)	1.22	0.954
Maximum occlusal force (N)	487.25±345.84	413.4±378.06	0.249	-73.846 (-288.434, 140.741)	104.22	437.76±282.77	435.05±270.54	0.733	-2.710 (-155.410, 149.990)	78.08	394.88±309.85	418.45±278.61	0.445	23.568 (-100.199, 147.335)	60.11	550.4±347.16	582.56±298.27	0.532	32.160 (-178.388, 242.708)	107.65	0.519
Tongue and lip motor function																					
/pa/ sound (times/s)	5.58±1.16	5.6±1.13	0.674	0.021 (-0.553, 0.596)	0.28	6.01±0.79	5.99±1.1	0.944	-0.020 (-0.568, 0.528)	0.28	5.47±1.08	5.42±1.29	0.812	-0.050 (-0.701, 0.601)	0.32	5.75±0.81	5.57±0.99	0.379	-0.180 (-0.848, 0.488)	0.34	0.478
/ta/ sound (times/s)	5.51±1.13	5.45±1.23	1.000	-0.064 (-0.643, 0.515)	0.28	5.91±0.65	5.94±0.65	0.573	0.033 (-0.268, 0.335)	0.15	5.4±1.13	5.48±1.2	0.440	0.079 (-0.428, 0.585)	0.25	5.61±0.99	5.61±0.93	0.283	0.000 (-0.408, 0.408)	0.21	0.779
/ka/ sound (times/s)	5.35±1.04	5.19±1.14	0.431	-0.164 (-0.732, 0.403)	0.28	5.44±0.68	5.56±0.64	0.277	0.123 (-0.360, 0.606)	0.25	5.03±1.02	4.97±1.18	0.953	-0.057 (-0.503, 0.388)	0.22	5.15±1.11	5.18±1.05	0.824	0.027 (-0.650, 0.704)	0.35	0.736
Maximum tongue pressure (kPa)	25.34±7.5	24.89±7.85	0.650	-0.450 (-7.114, 6.214)	3.24	24.8±10.32	24.37±9.87	0.691	-0.430 (-4.444, 3.584)	2.05	17.96±7.25	17.76±6.72	0.721	-0.200 (-2.784, 2.384)	1.26	21.56±9.34	25.57±9.73	0.394	4.010 (-4.119, 12.139)	4.16	0.095
Masticatory function (mg/dL)	209.39±95.28	243.82±120.76	0.173	34.423 (-48.426, 117.271)	40.24	227.3±106.98	227.6±69.39	0.865	0.300 (-84.502, 85.102)	43.36	185.09±69.31	257.39±134.86	0.074	72.298 (-41.691, 186.286)	55.36	222.63±64.58	255.86±106.24	0.496	33.228 (-60.874, 127.330)	48.11	0.554
EAT10	2.36±3.95	2.71±4.41	0.288	0.357 (-0.958, 1.672)	0.64	4.67±7.35	3.87±6.31	0.438	-0.800 (-5.507, 3.907)	2.41	6±6.46	4.36±6.4	0.028	-1.643 (-4.279, 0.993)	1.28	3.21±3.81	1.87±2.56	0.139	-1.214 (-4.046, 1.617)	1.38	0.521
RSST (times)	3.57±2.38	3.43±2.47	0.564	-0.143 (-1.120, 0.834)	0.47	2.8±1.82	2.87±2.1	0.928	0.067 (-1.799, 1.932)	0.95	3.14±1.29	3.5±1.51	0.160	0.357 (-0.599, 1.313)	0.46	2.87±1.81	2.87±1.77	1.000	0.000 (-1.109, 1.109)	0.57	0.618
**MNA**	23.86±3.24	23.64±3.51	0.721	-0.214 (-3.068, 2.639)	1.39	24.83±3.96	25.67±4.29	0.140	0.833 (-2.201, 0.368)	1.55	23.68±4.9	23.29±5.08	0.475	-0.393 (-2.267, 1.481)	0.91	24.03±3.89	23.9±3.75	0.806	-0.133 (-2.049, 1.782)	0.98	0.755
**Social frailty score**	1.5±0.65	1.36±0.63	0.317	0.143 (-0.693, 0.407)	0.27	1.6±1.12	1.47±1.46	0.596	-0.133 (-1.407, 1.140)	0.65	2.29±1.27	2.21±1.25	0.564	-0.071 (-0.560, 0.417)	0.24	2.53±1.46	2.33±1.35	0.380	-0.200 (-1.120, 0.720)	0.47	0.142
**GDS15**	3±2.32	3.43±3.16	0.472	0.429 (-1.697, 2.554)	1.03	3.8±2.93	3.87±4.02	0.543	0.067 (-2.255, 2.389)	1.19	4.36±3.59	4.57±2.79	0.478	0.214 (-1.507, 1.936)	0.84	5.87±4.5	5.53±4.52	0.561	-0.333 (-3.016, 2.349)	1.37	0.322

[*]: Wilcoxon signed-rank test at baseline and follow-up, [†]: Kruskal-wallis test at baseline for the four groups, [‡]: average±standard deviation, [§]:95% Confidence Interval, [‖]: 99.8% Confidence Interval

CI: Confidence Interval, MCID: minimum clinically important difference, BMI: Body mass index, SMI: skeletal mass index, EAT10: The 10-item Eating Assessment. Tool, RSST: Repetitive Saliva Swallowing Test, MNA: Mini Nutritional Assessment, GDS: Geriatric depression scale

Supplementary Table 3. Participants' characteristics at baseline (per-protocol analysis).

Table caption: This table summarizes baseline demographic, clinical, physical, oral function, and nutritional characteristics of participants in the four oral exercise frequency groups under the per-protcol analysis. Continuous variables are presented as mean ± standard deviation, and categorical variables as number (percentage). p-values are based on comparisons among groups. Bold variables indicate the main outcome measures: Number of frailty criteria, SMI, Number of present teeth, Number of functional teeth, Number of oral hypofunction criteria, MNA, Social frailty score, and GDS15.

	total		3 times/day & everyday (n=13)		3 times/day & 3 days/week (n=15)		once/day & everyday (n=10)		once/day & 3 days/week (n=15)		p value‡
Variables		95% CI		95% CI		95% CI		95% CI		95% CI	
age (y)	79.43±6.3*	(77.698, 81.17)	76.77±4.94	(73.787, 79.752)	78.87±5	(76.099, 81.635)	80.8±5.33	(76.988, 84.612)	81.4±8.42	(76.739, 86.061)	0.248
gender (Male)	12 (22.6)†	(5.0, 53.8)	3 (23.1)	(5.038, 53.813)	4 (26.7)	(7.787, 55.100)	2 (20.0)	(2.521, 55.610)	3 (20.0)	(4.331, 48.089)	0.971
**Number of frailty criteria**	2.15±0.95	(1.889, 2.412)	2.08±1.04	(1.450, 2.704)	2.13±0.83	(1.672, 2.595)	2.3±1.16	(1.471, 3.129)	2.13±0.92	(1.626, 2.64)	0.974
Grip strength (kg)	19.19±6.31	(17.453, 20.932)	19.27±4.22	(16.716, 21.822)	20.82±7.46	(16.69, 24.95)	16.93±7.66	(11.447, 22.413)	19.01±5.72	(15.841, 22.172)	0.478
Walking speed (m/s)	1.04±0.26	(0.965, 1.107)	1.05±0.23	(0.912, 1.194)	1.1±0.25	(0.956, 1.235)	1.01±0.29	(0.799, 1.214)	0.98±0.27	(0.83, 1.134)	0.548
**SMI (kg/m^2)**	6.17±0.78	(5.944, 6.390)	6.01±0.63	(5.616, 6.411)	6.34±0.8	(5.879, 6.799)	6.17±0.82	(5.586, 6.762)	6.12±0.91	(5.597, 6.645)	0.717
BMI (kg/m^2)	22.64±4.15	(21.493, 23.780)	21.46±5.29	(18.27, 24.658)	23.44±3.01	(21.777, 25.112)	21.94±3.38	(19.52, 24.351)	23.31±4.56	(20.786, 25.837)	0.377
Skeletal muscle mass	33.85±6.41	(32.082, 35.616)	32.56±5.14	(29.453, 35.662)	34.81±6.73	(31.087, 38.539)	34.14±7.04	(29.095, 39.175)	33.81±7.09	(29.886, 37.741)	0.799
Appendicular skeletal muscle mass	14.58±3.12	(13.691, 15.465)	13.98±2.56	(12.354, 15.604)	15.19±3.28	(13.291, 17.08)	14.96±3.76	(12.268, 17.652)	14.21±3.1	(12.422, 16)	0.756
Number of present teeth (n)	20±7.49	(17.936, 22.064)	20±7.77	(15.306, 24.694)	19.8±7.7	(15.535, 24.065)	21.1±5.57	(17.118, 25.082)	19.47±8.72	(14.635, 24.298)	0.998
Number of functional teeth (n)	26.43±3.38	(25.503, 27.365)	25.77±2.24	(24.415, 27.124)	27.07±2.15	(25.874, 28.259)	24.6±6.15	(20.201, 28.999)	27.6±2.1	(26.438, 28.762)	0.219
**Number of Oral hypofunction criteria (times)**	3.43±1.34	(3.065, 3.803)	3.15±0.99	(2.557, 3.75)	3.4±1.4	(2.622, 4.178)	3.8±1.99	(2.377, 5.223)	3.47±1.06	(2.88, 4.054)	0.747
Bacterial count on the dorsal surface of the tongue (10^6)(cfu/ml)	6.44±6.7	(4.589, 8.284)	5.92±5.88	(2.365, 9.47)	5.81±8.06	(1.348, 10.276)	8.38±7.71	(2.868, 13.9)	6.21±5.54	(3.146, 9.279)	0.474
Oral moisture status	25.69±3.62	(24.688, 26.684)	25.53±2.64	(23.932, 27.122)	25.06±5.45	(22.044, 28.076)	26.12±3.03	(23.951, 28.279)	26.16±2.52	(24.766, 27.561)	0.928
Maximum occlusal force (N)	470.23±316.6	(382.968, 557.500)	438.04±304.7	(253.907, 622.17)	437.76±282.77	(281.173, 594.354)	440.54±358.53	(184.065, 697.015)	550.4±347.16	(358.151, 742.656)	0.736
Tongue and lip motor function											
/pa/ sound (times/s)	5.72±0.95	(5.456, 5.982)	5.64±1.19	(4.921, 6.356)	6.01±0.79	(5.576, 6.45)	5.34±1.03	(4.604, 6.076)	5.75±0.81	(5.296, 6.197)	0.411
/ta/ sound (times/s)	5.62±0.96	(5.352, 5.879)	5.57±1.16	(4.869, 6.27)	5.91±0.65	(5.549, 6.265)	5.25±1.01	(4.526, 5.974)	5.61±0.99	(5.059, 6.154)	0.481
/ka/ sound (times/s)	5.23±0.95	(4.965, 5.490)	5.38±1.07	(4.727, 6.026)	5.44±0.68	(5.062, 5.811)	4.83±0.89	(4.193, 5.467)	5.15±1.11	(4.541, 5.765)	0.388
Maximum tongue pressure (kPa)	22.75±8.99	(20.274, 25.230)	24.81±7.53	(20.259, 29.364)	24.8±10.32	(19.086, 30.514)	18.79±7.55	(13.389, 24.191)	21.56±9.34	(16.388, 26.732)	0.288
Masticatory function (mg/dL)	211.93±89.07	(187.377, 236.481)	208.06±99.03	(148.215, 267.901)	227.3±106.98	(168.054, 286.546)	177.85±81.1	(119.837, 235.863)	222.63±64.58	(186.868, 258.398)	0.487
EAT10	3.69±5.17	(2.254, 5.131)	2.54±4.05	(0.089, 4.988)	4.67±7.35	(0.599, 8.734)	4.4±4.48	(1.197, 7.603)	3.21±3.81	(1.017, 5.412)	0.866
RSST	3.13±1.86	(2.619, 3.645)	3.69±2.43	(2.225, 5.16)	2.8±1.82	(1.792, 3.808)	3.3±1.06	(2.542, 4.058)	2.87±1.81	(1.866, 3.868)	0.536
**MNA**	24.41±3.37	(23.477, 25.335)	23.73±3.33	(21.717, 25.745)	24.83±3.96	(22.639, 27.028)	25.2±1.99	(23.777, 26.623)	24.03±3.63	(22.022, 26.045)	0.628
**Social frailty score**	1.92±1.21	(1.592, 2.257)	1.54±0.66	(1.139, 1.937)	1.6±1.12	(0.979, 2.221)	2±1.25	(1.108, 2.892)	2.53±1.46	(1.726, 3.34)	0.251
**GDS15**	4.17±3.54	(3.193, 5.147)	2.92±2.4	(1.475, 4.371)	3.8±2.93	(2.176, 5.424)	3.8±3.55	(1.258, 6.342)	5.87±4.5	(3.374, 8.36)	0.276
Stroke	1 (1.9)	(0.048, 10.070)	0 (0.0)	(0.000, 24.705)	0 (0.0)	(0.000, 21.802)	0 (0.0)	(0.000, 30.850)	1 (6.7)	(0.169, 31.948)	0.461
Heart disease	5 (9.4)	(3.135, 20.659)	1 (7.7)	(0.195, 36.030)	2 (13.3)	(1.658, 40.460)	1 (10.0)	(0.253, 44.502)	1 (6.7)	(0.169, 31.948)	0.929
Pulmonary disease	3 (5.7)	(2.095, 18.211)	3 (23.1)	(5.038, 53.813)	1 (6.7)	(0.169, 31.948)	0 (0.0)	(0.000, 30.850)	0 (0.0)	(0.000, 21.802)	0.088
Hypertension	24 (45.3)	(31.562, 59.550)	5 (38.5)	(13.858, 68.422)	5 (33.3)	(11.824, 61.620)	6 (60.0)	(26.238, 87.845)	8 (53.3)	(26.586, 78.733)	0.498
Diabetes mellitus	7 (13.2)	(5.479, 25.337)	5 (38.5)	(13.858, 68.422)	0 (0.0)	(0.000, 21.802)	0 (0.0)	(0.000, 30.850)	2 (13.3)	(1.658, 40.460)	0.012
Kidney disease	1 (1.9)	(0.048, 10.070)	1 (7.7)	(0.195, 36.030)	0 (0.0)	(0.000, 21.802)	0 (0.0)	(0.000, 30.850)	0 (0.0)	(0.000, 21.802)	0.371
Knee osteoarthritis	4 (7.5)	(2.095, 18.211)	1 (7.7)	(0.195, 36.030)	1 (6.7)	(0.169, 31.948)	1 (10.0)	(0.253, 44.502)	1 (6.7)	(0.169, 31.948)	0.989
Osteoporosis	10 (18.9)	(9.437, 31.972)	4 (30.8)	(9.092, 61.426)	1 (6.7)	(0.169, 31.948)	3 (30.3)	(6.674, 65.245)	2 (13.3)	(1.658, 40.460)	0.287
Rheumatoid arthritis	3 (5.7)	(1.183, 15.663)	1 (7.7)	(0.195, 36.030)	0 (0.0)	(0.000, 21.802)	1 (10.0)	(0.253, 44.502)	1 (6.7)	(0.169, 31.948)	0.710
Parkinson's disease	2 (3.8)	(0.460, 12.976)	0 (0.0)	(0.000, 24.705)	0 (0.0)	(0.000, 21.802)	1 (10.0)	(0.253, 44.502)	1 (6.7)	(0.169, 31.948)	0.473
Anemia	4 (7.5)	(2.095, 18.211)	1 (7.7)	(0.195, 36.030)	2 (13.3)	(1.658, 40.460)	0 (0.0)	(0.000, 30.850)	1 (6.7)	(0.169, 31.948)	0.670
Hearing dysfunction	1 (1.7)	(0.048, 10.070)	0 (0.0)	(0.000, 24.705)	1 (6.7)	(0.169, 31.948)	0 (0.0)	(0.000, 30.850)	0 (0.0)	(0.000, 21.802)	0.461

*: average±standard deviation, †: n (%), ‡: p-values for group comparisons using Kruskal–Wallis test for continuous variables and chi-square test for categorical variables

CI: Confidence Interval, BMI: Body mass index, SMI: skeletal mass index, EAT10: The 10-item Eating Assessment. Tool, RSST: Repetitive Salive Swallowing Test, MNA: Mini Nutritional Assessment, GDS: Geriatric depression scale

Supplementary Table 4. The baseline and follow-up outcomes and minimum clinically important difference in the each groups (per-protocol analysis).

Table caption: This table presents baseline and follow-up values for frailty criteria, physical performance, sarcopenia-related measures, oral function, and nutritional status in four oral exercise frequency groups under the per-protocol analysis. It also includes p-values, mean differences with 95% confidence intervals, and minimum clinically important differences (MCID) for each outcome.

	3 times/day & everyday (n=13)					3 times/day & 3 days/week (n=15)					once/day & everyday (n=9)					once/day & 3 days/week (n=15)					p-value[†]
	baseline	Follow-Up	p-value*	Mean difference (CI)	MCID	baseline	Follow-Up	p-value	Mean difference (CI)	MCID	baseline	Follow-Up	p-value	Mean difference (CI)	MCID	baseline	Follow-Up	p-value	Mean difference (CI)	MCID	
**Number of frailty criteria**	2.08±1.04‡	1.69±0.95	0.096	-0.385 (-0.849, 0.079)[§]	0.38	2.13±0.83	1.53±0.99	0.070	-0.600 (-1.255, 0.055)	0.59	2.3±1.16	1.5±0.97	0.155	-0.800 (-1.958, 0.358)	0.81	2.13±0.92	1.53±1.25	0.013	-0.600 (-1.008, -0.192)	0.37	0.974
Grip strength (kg)	19.27±4.22	19.58±3.3	0.345	0.315 (-2.433, 3.064)[‖]	1.26	20.82±7.46	21.09±7.66	0.755	0.273 (-1.949, 2.496)	1.14	16.93±7.66	17.72±8.14	0.414	0.790 (-2.498, 4.078)	1.21	19.01±5.72	18.55±5.04	0.211	-0.453 (-2.198, 1.292)	0.89	0.478
Walking speed (m/s)	1.05±0.23	1.06±0.35	0.917	0.007 (-0.183, 0.197)	0.09	1.1±0.25	1.15±0.47	0.460	0.051 (-0.249, 0.350)	0.15	1.01±0.29	0.98±0.2	0.799	-0.024 (-0.265, 0.217)	0.09	0.98±0.27	1.02±0.38	0.280	0.035 (-0.292, 0.362)	0.17	0.548
**SMI (kg/m^2)**	6.01±0.63	6.02±0.48	0.308	-0.018 (-0.421,0.385)	0.17	6.34±0.8	6.44±0.71	0.152	0.089 (-0.155, 0.333)	0.11	6.17±0.82	6.14±0.81	0.508	-0.035 (-0.220, 0.149)	0.07	6.12±0.91	6.2±0.75	0.730	0.021 (-0.458, 0.499)	0.23	0.717
BMI (kg/m^2)	21.46±5.29	21.25±5.25	0.055	-0.218 (-0.926, 0.491)	0.33	23.44±3.01	23.49±2.99	0.691	0.042 (-0.686, 0.769)	0.37	21.94±3.38	21.61±3.18	0.047	-0.323 (-0.871, 0.225)	0.20	23.31±4.56	23.23±4.44	0.650	-0.081 (-0.614, 0.452)	0.27	0.377
Skeletal muscle mass	32.56±5.14	32.6±4.83	0.824	0.046 (-1.184, 1.276)	0.56	34.81±6.73	35.19±6.16	0.382	0.021 (-1.510, 1.553)	0.74	34.14±7.04	34.02±7.06	0.646	-0.120 (-0.928, 0.688)	0.30	33.81±7.09	33.8±6.51	0.842	-0.013 (-1.423, 1.397)	0.72	0.799
Appendicular skeletal muscle mass	13.98±2.56	14.01±2.11	0.455	-0.054 (-0.957, 0.849)	0.39	15.19±3.28	15.45±3.04	0.124	0.196 (-0.526, 0.918)	0.33	14.96±3.76	14.9±3.77	0.678	-0.065 (-0.591, 0.461)	0.19	14.21±3.1	14.31±2.58	0.730	-0.100 (-1.382, 1.182)	0.62	0.756
**Number of present teeth (n)**	20±7.77	19.92±7.84	0.317	-0.077 (-0.379, 0.225)	0.14	19.8±7.7	19.8±7.7	1.000	0.000 (0.000, 0.000)	0.00	21.1±5.57	21.1±5.57	1.000	0.000 (0.000, 0.000)	0.00	19.47±8.72	19.47±8.72	1.000	0.000 (0.000, 0.000)	0.00	0.998
**Number of functional teeth (n)**	25.77±2.24	25.69±2.18	0.317	-0.077 (-0.379, 0.225)	0.14	27.07±2.15	27.07±2.15	1.000	0.000 (0.000, 0.000)	0.00	24.6±6.15	24.6±6.15	1.000	0.000 (0.000, 0.000)	0.00	27.6±2.1	27.6±2.1	1.000	0.000 (0.000, 0.000)	0.00	0.219
**Number of Oral hypofunction criteria**	3.15±0.99	2.62±1.12	0.070	-0.538 (-1.593, 0.516)	0.48	3.4±1.4	3.07±1.49	0.374	-0.333 (-1.596, 0.929)	0.65	3.8±1.99	2.9±1.79	0.084	-0.900 (-2.869, 1.069)	0.72	3.47±1.06	2.64±1.08	0.005	-0.929 (-1.955, 0.098)	0.50	0.747
Bacterial count on the dorsal surface of the tongue (10^6)(cfu/ml)	5.92±5.88	5.77±4.53	0.969	-0.150 (-4.477, 4.178)	1.99	5.81±8.06	6.49±7.97	0.600	0.679 (-6.524, 7.882)	3.68	8.38±7.71	12.4±8.14	0.093	4.011 (-8.559, 16.581)	4.63	6.21±5.54	11.63±16.35	0.307	5.417 (-9.235, 20.068)	7.49	0.474
Oral moisture status	25.53±2.64	28.91±0.85	0.005	3.381 (0.374, 6.387)	1.38	25.06±5.45	27.97±2.15	0.088	2.910 (-2.859, 8.679)	2.95	26.12±3.03	28.81±2.3	0.047	2.690 (-2.005, 7.385)	1.73	26.16±2.52	29.19±2.01	0.003	3.030 (0.646, 5.414)	1.22	0.928
Maximum occlusal force (N)	438.04±304.7	358.51±330.36	0.249	-79.527 (-314.748, 155.694)	107.91	437.76±282.77	435.05±270.54	0.733	-2.710 (-155.410, 149.990)	78.08	440.54±358.53	473.54±313.51	0.445	32.995 (-161.701, 227.691)	71.64	550.4±347.16	582.56±298.27	0.532	32.160 (-178.388, 242.708)	107.65	0.736
Tongue and lip motor function																					
/pa/ sound (times/s)	5.64±1.19	5.66±1.15	0.674	0.023 (-0.610, 0.656)	0.29	6.01±0.79	5.99±1.1	0.944	-0.020 (-0.568, 0.528)	0.28	5.34±1.03	5.27±1.33	0.812	-0.070 (-1.101, 0.961)	0.38	5.75±0.81	5.57±0.99	0.379	-0.180 (-0.848, 0.488)	0.34	0.411
/ta/ sound (times/s)	5.57±1.16	5.5±1.26	1.000	-0.069 (-0.707, 0.568)	0.29	5.91±0.65	5.94±0.65	0.573	0.033 (-0.268, 0.335)	0.15	5.25±1.01	5.36±1.13	0.440	0.110 (-0.689, 0.909)	0.29	5.61±0.99	5.61±0.93	0.283	0.000 (-0.408, 0.408)	0.21	0.481
/ka/ sound (times/s)	5.38±1.07	5.2±1.19	0.431	-0.177 (-0.800, 0.446)	0.29	5.44±0.68	5.56±0.64	0.277	0.123 (-0.360, 0.606)	0.25	4.83±0.89	4.75±1.13	0.953	-0.080 (-0.784, 0.624)	0.26	5.15±1.11	5.18±1.05	0.844	0.027 (-0.650, 0.704)	0.35	0.388
Maximum tongue pressure (kPa)	24.81±7.53	24.33±7.88	0.650	-0.485 (-7.826, 6.856)	3.37	24.8±10.32	24.37±9.87	0.691	-0.430 (-4.444, 3.584)	2.05	18.79±7.55	18.51±6.84	0.721	-0.280 (-4.374, 3.814)	1.51	21.56±9.34	25.57±9.73	0.394	4.010 (-4.119, 12.139)	4.16	0.288
Masticatory function (mg/dL)	208.06±99.03	245.13±125.58	0.173	37.071 (-53.526, 127.667)	41.56	227.3±106.98	227.6±69.39	0.865	0.300 (-84.502, 85.102)	43.36	177.85±81.1	279.07±155.84	0.074	101.217 (-62.154, 264.587)	60.12	222.63±64.58	255.86±106.24	0.496	33.228 (-60.874, 127.330)	48.11	0.487
EAT10	2.54±4.05	2.92±4.52	0.288	0.385 (-1.060, 1.829)	0.66	4.67±7.35	3.87±6.31	0.438	-0.800 (-5.507, 3.907)	2.41	4.4±4.48	2.1±3.03	0.028	-2.300 (-6.092, 1.492)	1.40	3.21±3.81	1.87±2.56	0.139	-1.214 (-4.046, 1.617)	1.38	0.866
RSST (times)	3.69±2.43	3.54±2.54	0.564	-0.154 (-1.230, 0.922)	0.49	2.8±1.82	2.87±2.1	0.928	0.067 (-1.799, 1.932)	0.95	3.3±1.06	3.8±1.32	0.160	0.500 (-0.968, 1.968)	0.54	2.87±1.81	3.07±1.64	0.813	0.071 (-1.103, 1.246)	0.57	0.536
**MNA**	23.73±3.33	23.5±3.61	0.721	-0.231 (-3.374, 2.912)	1.44	24.83±3.96	25.67±4.29	0.140	0.833 (-2.201, 3.868)	1.55	25.2±1.99	24.65±2.88	0.475	-0.550 (-3.493, 2.393)	1.08	24.03±3.63	23.9±3.75	0.806	-0.133 (-2.049, 1.782)	0.98	0.623
**Social frailty score**	1.54±0.66	1.38±0.65	0.317	-0.154 (-0.758, 0.451)	0.28	1.6±1.12	1.47±1.46	0.596	-0.133 (-1.407, 1.140)	0.65	2±1.25	1.9±1.2	0.564	-0.100 (-0.871, 0.671)	0.28	2.53±1.46	2.33±1.35	0.380	-0.200 (-1.120, 0.720)	0.47	0.251
**GDS15**	2.92±2.4	3.38±3.28	0.472	0.462 (-1.877, 2.800)	1.07	3.8±2.93	3.87±4.02	0.543	0.067 (-2.255, 2.389)	1.19	3.8±3.55	4.1±2.38	0.478	0.300 (-2.421, 3.021)	1.00	5.87±4.5	5.53±4.52	0.561	-0.333 (-3.016, 2.349)	1.37	0.276

[*]: Wilcoxon signed-rank test at baseline and follow-up, [†]: Kruskal-wallis test at baseline for the four groups, [‡]: average±standard deviation, [§]:95% Confidence Interval, [‖]: 99.8% Confidence Interval

CI: Confidence Interval, MCID: minimum clinically important difference, BMI: Body mass index, SMI: skeletal mass index, EAT10: The 10-item Eating Assessment. Tool, RSST: Repetitive Saliva Swallowing Test, MNA: Mini Nutritional Assessment, GDS: Geriatric depression scale

Supplementary Table 5. Results of two-way repeated measures ANOVA analysis in 4 groups (per-protocol analysis).

Table caption: This table summarizes mean differences (95% confidence intervals) from baseline to follow-up for frailty criteria, sarcopenia-related measures, oral hypofunction, and other outcomes across four oral exercise frequency groups under the per-protocol analysis. It also presents F-values, p-values, and partial η² for the main effect of time and the interaction between group and time.

	Mean difference (CI)				Time			Group x Time		
	3 times/day & everyday	3 times/day & 3 days/week	once/day & everyday	once/day & 3 days/week	F	p	ηp^2	F	p	ηp^2
Number of frailty criteria	-0.385 (-0.849, 0.079)*	-0.467 (-0.974, 0.040)	-1.000 (-1.769, -0.231)	-0.733 (-1.266, -0.201)	0.020	0.996	0.001	0.280	0.840	0.017
SMI (kg/m^2)	-0.018 (-0.349, 0.313)†	0.089 (-0.112, 0.291)	-0.035 (-0.185, 0.114)	0.021 (-0.377, 0.418)	0.701	0.556	0.045	0.338	0.798	0.022
Number of present teeth (n)	-0.077 (-0.327, 0.173)	0.000 (0.000, 0.000)	0.000 (0.000, 0.000)	0.000 (0.000, 0.000)	0.095	0.962	0.006	1.027	0.389	0.059
Number of functional teeth (n)	-0.077 (-0.327, 0.173)	0.000 (0.000, 0.000)	0.000 (0.000, 0.000)	0.000 (0.000, 0.000)	2.067	0.117	0.112	1.027	0.389	0.059
Number of Oral hypofunction criteria (times)	-0.538 (-1.410, 0.333)	-0.333 (-1.386, 0.719)	-0.900 (-2.492, 0.692)	-0.929 (-1.781, -0.076)	0.316	0.814	0.019	0.814	0.493	0.048
MNA	-0.231 (-2.828, 2.366)	0.833 (-1.696, 3.363)	-0.550 (-2.930, 1.830)	-0.133 (-1.730, 1.463)	0.729	0.540	0.043	0.708	0.552	0.042
Social frailty score	-0.154 (-0.653, 0.346)	-0.133 (-1.194, 0.928)	-0.100 (-0.724, 0.524)	-0.200 (-0.967, 0.567)	2.395	0.080	0.128	0.026	0.994	0.002
GDS15	0.462 (-1.470, 2.393)	0.067 (-1.869, 2.002)	0.300 (-1.900, 2.500)	-0.333 (-2.569, 1.902)	1.450	0.240	0.082	0.292	0.831	0.018

ηp2: partial eta squared as a measure of the effect size.

CI: Confidence interval, SMI: skeletal mass index, MNA: Mini Nutritional Assessment, GDS: Geriatric depression scale

*: 95% Confidence interval, †: 99.3% Confidence interval

Supplementary Table 6. Results of generalized linear model in 4 groups (intention-to-treat analysis).

Table caption: Results of generalized linear model (two-sided) for frailty, EAT-10, social frailty, and GDS-15 scores under the intention-to-treat analysis. Multiple comparisons were adjusted using the Bonferroni correction.

variables		odds ratio	95%CI	p value	Time	Group x Time
					p value	p value
Frailty[1)]	3 times/day & everyday	2.649	0.488-14.371	0.259	0.002	0.642
	3 times/day & 3 days/week	1.000	0.189-5.299	1.000		
	once /day & everyday	3.646	0.675-19.696	0.133		
	once /day & 3 days/week	ref				
EAT-10[2)]	3 times/day & everyday	1.250	0.257-6.070	0.782	0.171	0.749
	3 times/day & 3 days/week	1.000	0.219-4.564	1.000		
	once /day & everyday	0.900	0.194-4.165	0.893		
	once /day & 3 days/week	ref				
Social frailty[3)]	3 times/day & everyday	0.108	0.022-0.543	0.007	0.745	0.990
	3 times/day & 3 days/week	0.148	0.027-0.793	0.026		
	once /day & everyday	0.545	0.124-2.396	0.422		
	once /day & 3 days/week	ref				
GDS15[4)]	3 times/day & everyday	0.622	0.071-1.461	0.142	0.260	0.588
	3 times/day & 3 days/week	0.434	0.101-1.856	0.260		
	once /day & everyday	0.634	0.161-2.501	0.515		
	once /day & 3 days/week	ref				

EAT-10: EAT10: The 10-item Eating Assessment Tool, GDS: Geriatric depression scale

[1)] 0: robust, 1: pre-frailty, 2: frailty

[2)] 0: none, 1: dysphagia

[3)] 0: robust, 1: pre-frailty, 2: frailty

[4)] 0: none, 1: mild depression, 2: severe depression

Supplementary Table 7. Results of generalized linear model in 4 groups (per-protocol analysis).

Table caption: Results of generalized linear model (two-sided) for frailty, EAT-10, social frailty, and GDS-15 scores under the per-protocol (PP) analysis. Multiple comparisons were adjusted using the Bonferroni correction.

variables		odds ratio	95%CI	p value	Time	Group x Time
					p value	p value
Frailty[1)]	3 times/day & everyday	0.754	0.496-15.289	0.247	0.002	0.793
	3 times/day & 3 days/week	1.000	0.191-5.242	1.000		
	once /day & everyday	2.181	0.343-13.880	0.409		
	once /day & 3 days/week	ref				
EAT10[2)]	3 times/day & everyday	1.125	0.229-5.537	0.885	0.105	0.563
	3 times/day & 3 days/week	1.000	0.219-4.564	1.000		
	once /day & everyday	2.000	0.304-13.173	0.471		
	once /day & 3 days/week	ref				
Social frailty[3)]	3 times/day & everyday	0.110	0.022-0.564	0.008	0.71	0.987
	3 times/day & 3 days/week	0.152	0.029-0.807	0.027		
	once /day & everyday	0.389	0.072-2.097	0.272		
	once /day & 3 days/week	ref				
GDS15[4)]	3 times/day & everyday	0.365	0.080-1.665	0.193	0.232	0.567
	3 times/day & 3 days/week	0.442	0.104-1.875	0.268		
	once /day & everyday	0.641	0.144-2.850	0.559		
	once /day & 3 days/week	ref				

EAT-10: EAT10: The 10-item Eating Assessment Tool, GDS: Geriatric depression scale

[1)] 0: robust, 1: pre-frailty, 2: frailty

[2)] 0: none, 1: dysphagia

[3)] 0: robust, 1: pre-frailty, 2: frailty

[4)] 0: none, 1: mild depression, 2: severe depression
